# *Notes from the Field:* Recurrence of a Multistate Outbreak of *Salmonella* Enteritidis Infections Linked to Contact with Guinea Pigs — Eight States, 2015–2017

**DOI:** 10.15585/mmwr.mm6742a6

**Published:** 2018-10-26

**Authors:** Scott Robertson, Alexis Burakoff, Lauren Stevenson, Bradley Tompkins, Kane Patel, Beth Tolar, Laura Whitlock, Jennifer House, Linda Schlater, Tonya Mackie, Brenda Morningstar-Shaw, Megin Nichols, Colin Basler

**Affiliations:** ^1^Epidemic Intelligence Service, CDC; ^2^Division of Foodborne, Waterborne and Environmental Diseases, National Center for Emerging and Zoonotic Infectious Diseases, CDC; ^3^Colorado Department of Public Health and Environment; ^4^Vermont Department of Health; ^5^National Veterinary Services Laboratories, U.S. Department of Agriculture, Ames, Iowa; ^6^CAITTA, Inc., Herndon, Virginia.

In December 2017, the Colorado Department of Public Health and Environment reported two human *Salmonella* Enteritidis infections in persons with exposure to pet guinea pigs. The guinea pigs had been purchased from two separate pet stores, belonging to a single chain, and supplied by a common distributor located in California. Clinical isolates were indistinguishable by pulsed-field gel electrophoresis (PFGE), suggesting the infections were related. This PFGE pattern was previously seen in a 2010 multistate outbreak linked to contact with pet guinea pigs (*1*). An investigation was initiated to determine the number of patients affected and to identify the source of human illnesses.

A case was defined as *Salmonella* Enteritidis infection with a clinical isolate having an identical PFGE pattern to those from the Colorado isolates and closely related to a guinea pig isolate by whole genome sequencing (WGS), and with onset of clinical signs on or after January 1, 2015. State health departments were asked to review recent *Salmonella* Enteritidis illness records for patient exposure to guinea pigs. In addition, the U.S. Department of Agriculture’s National Veterinary Services Laboratories was queried for isolates from guinea pigs that matched the outbreak strain. All isolates underwent WGS using high-quality single nucleotide polymorphism (SNP) analysis. An isolate from the 2010 outbreak was sequenced for comparison. Guinea pig purchase invoices were used to trace guinea pigs with an epidemiologic link to human illness back to the distributor of origin.

Nine cases in humans were identified from eight states, including two cases in Colorado and one each in Iowa, Indiana, Massachusetts, Michigan, New York, Vermont, and Virginia. Five of eight patients reported exposure to guinea pigs. Onset dates ranged from July 15, 2015, to December 15, 2017. The median patient age was 12 years (range = 1–70 years). Five patients were female. One patient was hospitalized, and no deaths were reported. Six isolates submitted to veterinary diagnostic laboratories from ill guinea pigs and one isolate from a patient’s guinea pig were sequenced and found to be closely related to the outbreak strain. Including the 2010 isolate tested for comparison, all isolates were within 38 SNPs by WGS.

Traceback information was available for four guinea pigs purchased from two large pet store chains (Figure). The two distributors supplying guinea pigs to pet stores during this outbreak received guinea pigs from multiple wholesalers; however, a single common wholesaler was mentioned by both. This wholesaler also supplied guinea pigs that were associated with cases during the 2010 outbreak.

**FIGURE:**
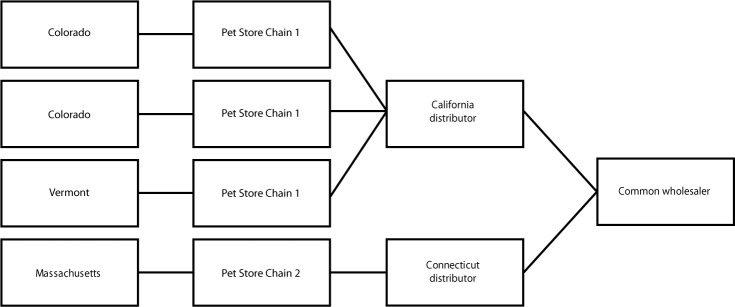
Traceback* of guinea pigs associated with human salmonellosis from patient to distributor of origin (n = 4) — three states, 2015–2017 * Traceback for guinea pig distribution from patients back to a common wholesaler. The Colorado and Vermont patients purchased guinea pigs from Pet Store Chain 1, which received guinea pigs from a California distributor. The Massachusetts patient purchased a guinea pig from Pet Store Chain 2, which received guinea pigs from a Connecticut distributor. Both distributors had a single common wholesaler.

Following the 2010 outbreak, recommendations including environmental testing were made to the wholesaler regarding *Salmonella* prevention; however, the actions were not implemented. Failure to implement recommended prevention measures might have contributed to recurrence of the outbreak. To enhance compliance with recommendations made in this outbreak, CDC developed a document containing prevention measures aimed at reducing the prevalence of *Salmonella* in guinea pig colonies intended for use in the pet industry. Content was also posted on the CDC website to increase consumer awareness of risk for *Salmonella* infection linked to pet guinea pigs. Recommendations to pet owners during this outbreak focused on proper hand hygiene. Recommendations to distributors and wholesalers included routine monitoring of guinea pigs for *Salmonella* through diagnostic testing, recordkeeping to aid in traceback, and evaluating husbandry and environmental sanitation practices of guinea pig breeders to reduce the prevalence of *Salmonella* and other zoonotic diseases of concern to the pet industry (*2*).
